# Impact of standardising the colour and branding of vape devices on product appeal among young people: a randomised experiment in England, Canada and the United States

**DOI:** 10.1136/tc-2024-059210

**Published:** 2025-05-20

**Authors:** Harry Tattan-Birch, Katherine East, Sharon Cox, Sarah Jackson, Jamie Brown, Erikas Simonavicius, Jessica L Reid, David Hammond, Eve Violet Taylor

**Affiliations:** 1 Department of Behavioural Science and Health, University College London, London, UK; 2 Department of Primary Care and Public Health, Brighton and Sussex Medical School, University of Brighton and University of Sussex, Brighton, UK; 3 Department of Addictions, Institute of Psychiatry, Psychology and Neuroscience, King’s College London, London, UK; 4 School of Public Health Sciences, University of Waterloo, Waterloo, Ontario, Canada

**Keywords:** Packaging and Labelling, Prevention, Electronic nicotine delivery devices, Advertising and Promotion

## Abstract

**Objective:**

To estimate the impact of standardising the colour and branding of disposable vaping devices on young people’s interest in trying them.

**Design, setting and participants:**

Data were from national surveys of 16–29-year-olds in Canada, England and the United States in 2023 (N=15 259).

**Interventions:**

Respondents were randomised (1:1) to view images of either four branded disposable vapes (N=7638) or four standardised white disposable vapes (n=7621) and asked which they would be interested in trying.

**Main outcome measures:**

The primary outcome was selecting “no interest in trying” rather than any of the vapes displayed. We also examined whether the impact of standardisation differed by five potential moderators.

**Results:**

A greater proportion of participants reported “no interest in trying” the white standardised than branded vapes (67.1% vs 62.8%; adjusted risk ratio (ARR) 1.127, 95% CI 1.085 to 1.169). Compared with those who had never smoked or vaped, the impact of standardisation on those reporting no interest was greater for those who had, in the past 30 days, only smoked (47.5% vs 37.5%, ARR 1.287, 95% CI 1.079 to 1.495), only vaped (19.9% vs 16.4%, ARR 1.220, 95% CI 1.002 to 1.438), dual used (13.5% vs 9.5%, ARR 1.420, 95% CI 1.017 to 1.822) or who had formerly vaped/smoked (72.6% vs 65.0%, ARR 1.119, 95% CI 1.071 to 1.167). The impact was also greatest in the oldest age group, but there were no other clear moderators.

**Conclusions:**

Standardising the colour and branding of disposable vaping devices reduces young people’s interest in trying them. However, this includes a substantial impact on those who smoke.

**WHAT IS ALREADY KNOWN ON THIS TOPIC**

- Tobacco and vape packaging that is standardised is less appealing to young people, but the effect of standardising the colour and branding of the vape device itself is unknown.

**WHAT THIS STUDY ADDS**

- Standardising the colour and branding of disposable vape devices reduces young people’s interest in trying them, but this includes a large effect among people who smoke.

**HOW THIS STUDY MIGHT AFFECT RESEARCH, PRACTICE OR POLICY**

- Trade-offs must be considered, as while standardisation of vape devices likely deters young people who have never smoked from trying them, it may also lead to fewer people switching from smoking to vaping.

## Background

 E-cigarette use (‘vaping’) has risen rapidly among youth and young adults in England, Canada and the United States (US) over the past decade (with the US seeing recent reductions).^1–3^ The rise in vaping has coincided with the introduction of disposable devices, which are most commonly used among young people,^4–6^ and increasingly among those who have never smoked regularly.^7 8^ Disposable devices are more commonly used for those experimenting with vaping than other devices,^9^ although they are increasingly being used long term.^8^

Although vapes can help people quit smoking,^10^ it is possible that the design, packaging and marketing of the new disposable products are attractive to, and encourage use among, young people who do not smoke.^8 11 12^ New measures to reduce youth vaping would ideally reflect this trade-off and reduce appeal among never-smokers without deterring or misleading smokers into believing vapes are more harmful than cigarettes (note that only a minority of young people who smoke perceive vaping to be less harmful than smoking).^13 14^ Product and packaging regulations have previously been used by many countries to reduce the appeal of cigarettes.^15 16^ For example, in the UK, cigarettes must be white, with a white or cork tip, and be sold in standardised green packaging.^17^ Brand names are allowed on sticks in a specified position, size and font, but all other trademarks, logos, colour schemes and graphics are prohibited.^17^ Standardised packaging for e-cigarettes is currently required in Israel^18^ (standardised to brown/green) and the Netherlands^19^ (standardised to a neutral colour), and is being considered by the UK government.^20^ However, there is little regulation globally on the design of the actual vaping device.

Previous experiments have found that removing brand imagery and brand names from e-cigarette packaging can reduce appeal to youth without reducing appeal to adults who smoke.^21–23^ Unlike cigarettes, e-cigarette packaging is usually discarded immediately after first opening it. Therefore, the design of the device itself is an important marketing feature for e-cigarette brands. Colourful “skins” are also available for some e-cigarette devices, which may also increase appeal.^23^ Earlier work on cigarette packaging suggests youth and young adults perceive colourful, patterned and slim cigarettes as appealing and less harmful than other cigarettes.^24 25^ However, there is no current evidence on whether standardising the colour and branding of e-cigarette devices themselves affects their appeal and perceptions of harm to young people, or whether this effect differs across people from various sociodemographic groups and by vaping/smoking status.

### Research questions

Our primary research questions were:

1. What is the impact of standardising the colour and branding of disposable vaping devices on interest in trying them among young people (aged 16–29 years)?
2. What is the impact of standardising the colour and branding of disposable vaping devices on vaping harm perceptions among young people (aged 16–29 years)?

We also explored the following:

1. Does the impact of standardisation on interest in trying vaping and harm perceptions differ by age groups (16–17, 18–19 or 20–29 years old), sex, vaping/smoking status, perceived family income adequacy, or country (England, Canada, US)?

### Hypotheses

1. Young people will be less likely to report interest in trying standardised white vaping devices than branded vaping devices in their original colours.
2. Young people will be less likely to perceive standardised white vaping devices as less harmful than cigarettes compared with branded vaping devices in their original colours.

We had no specific a priori hypotheses for research question 3, so the analysis of potential moderators should be considered exploratory.

## Methods

### Design and participants

This study was a randomised experiment whereby participants were randomly allocated (at a 1:1 ratio) to either the branded or standardised condition, stratified by and balanced within each country (ie, randomised without replacement, with randomisation sequence calculated via the survey programming software). This experiment was embedded within the 2023 wave of the online ITC Youth and Youth Adult Tobacco & Vaping Survey. Thus, the sample size was determined based on the number of participants recruited in this survey, rather than planned specifically for this experiment. Technical reports are available online (https://davidhammond.ca/projects/tobacco-vaping/itc-youth-tobacco-ecig/).

Participants were aged between 16 and 29 years old, residing in England, Canada or the US. Participants were recruited via the Nielsen Consumer Insights Global Panel and partner panels. Participants received remuneration in accordance with their panel’s usual incentive structure, which could include points-based or monetary rewards (redeemed for catalogue items, as cash or donated) and/or opportunities to win monthly prizes.

### Procedures

**Figure 1:**
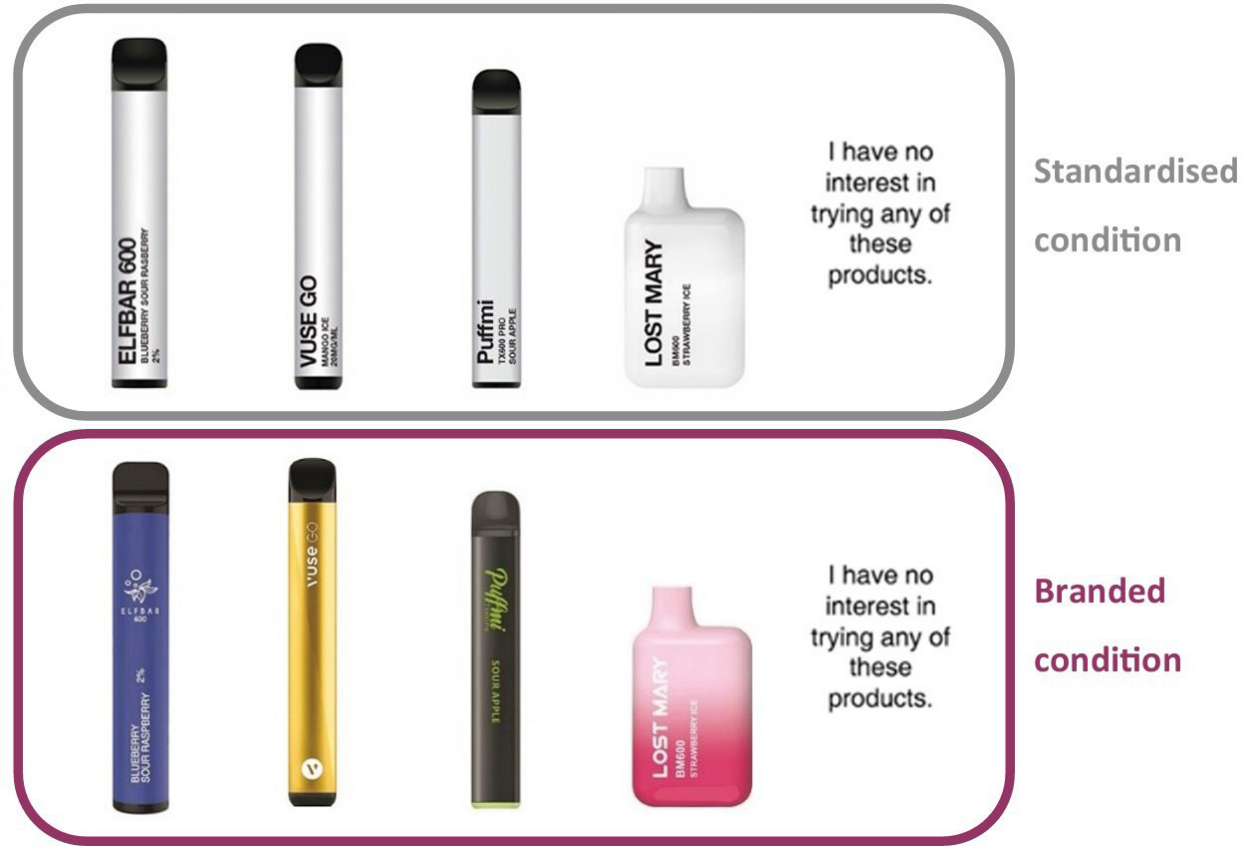
The branded and standardised vaping devices shown to participants. Participants were randomised to view either all four standardised devices (experimental condition) *or* all four branded devices (control condition).

Participants were randomly allocated to either a standardised (experimental) condition or branded (control) condition. Those randomised to the standardised condition viewed a set of four images of vaping devices with standardised colour (white) and text (brand name and flavour in uniform typeface (eg, upper images in figure 1). Those in the branded condition viewed a set of four images of vaping devices with branding and colours as they are currently sold in England (eg, lower images in figure 1). Then, they were shown one of the four vapes within their condition at random and asked to rate its harmfulness (see specific wording in ‘Outcome measures’ section).

Products from popular brands were chosen for the experiment,^1^ with a range of colours to partially reflect the array of disposable products currently available on the market. Fruit flavours were chosen as they are the most popular among youth.^26^

Participants were assigned to either only view standardised devices or only view branded devices for two reasons. First, this prevents contamination effects that could occur if participants were exposed to both standardised and branded vapes simultaneously. Second, this meant the experiment more closely captured the choice that an individual would make if regulations were introduced, where all vapes on display would be standardised.

A standardised colour of white was selected because it is more neutral than most branding but distinct from the green/brown (Pantone 448 C) required for tobacco packaging in many countries including England and Canada, thereby intending to reflect risk differences in the use of cigarettes and vapes.

### Outcome measures

*Interest in trying*. Respondents were asked “Which of the following vapes would you be interested in trying?” with options to select any of the four vapes displayed within each condition (including multiple) with response options “I have no interest in trying any of these products”, “Don’t know” or “Refused”. For the primary analyses, this was coded as no interest in trying versus otherwise (ie, selected at least one of the vapes displayed or “Don’t know”). In a sensitivity analysis, we used the following ordinal scale, where higher scores indicate greater interest: 1=No interest in trying, 2=“Don’t know”, 3=Selected one device, 4=Selected two devices, 5=Selected three devices, 6=Selected all four devices. In both analyses, we excluded individuals who selected “Refused”.

*Harm perceptions*. Participants were shown one of the products within their condition at random and were asked “How harmful do you think it is to vape this product?” with response options “Not at all harmful”, “Harmful, but less harmful than smoking cigarettes”, “As harmful as smoking cigarettes”, “More harmful than smoking cigarettes”, “Don’t know” or “Refused”. Current evidence suggests that vaping is less harmful than smoking,^27 28^ so in the primary analysis responses were dichotomised as less harmful than smoking (ie, “Not at all harmful” or “Harmful, but less harmful than smoking cigarettes”) versus otherwise (including “Don’t know” but excluding “Refused”). In a sensitivity analysis, we dichotomised responses as those who perceived e-cigarettes as “Not at all harmful” versus otherwise (excluding “Refused”) to examine whether standardisation affected the proportion of participants who inaccurately viewed vaping as entirely harmless.

### Sample characteristics

The full questionnaire is available online.^29^

*Age group*. Current age in years was categorised into three groups (16–17, 18–19 and 20–29 years).

*Sex at birth*. Sex at birth (male/female) was reported by participants, or inferred from reported gender in the small number of cases where male or female sex at birth was not specified.

*Race/ethnicity*. Participants were asked about their race/ethnicity using country-specific measures based on Census questions in England, Canada and the US. For a common measure, we collapsed country-specific options to examine differences between White (the majority group) and other groups (other and mixed ethnicities, “Don’t know” and “Refused”).^30^ Sample sizes did not permit detailed analysis of specific minority ethnic groups.

*Past 30-day vaping/smoking status*. Participants were asked separate sets of questions about smoking cigarettes and using e-cigarettes/vaping, beginning with “Have you ever tried [an e-cigarette/vaped OR cigarette smoking], even one or two puffs?”. Those who responded “Yes” were then asked “When was the last time you [used an e-cigarette/vaped OR smoked a cigarette], even one or two puffs?”. Responses were categorised into five mutually exclusive use subgroups, aligning with previous studies^21^: (1) past 30-day exclusive vaping (ie, vaped but did not smoke in the past 30 days); (2) past 30-day exclusive smoking (ie, smoked but did not vape in the past 30 days); (3) past 30-day use (ie, both vaped and smoked in the past 30 days); (4) ever but not past 30-day vaping and/or smoking (ie, ever vaped and/or smoked, but not in the past 30 days) and (5) never vaped or smoked (never vaped and never smoked).

*Perceived family income adequacy*. Participants were asked “How would you describe your family’s financial situation?”. Responses were ordered on a four-point scale: 0=“Not meeting basic expenses”, 1=“Just meeting basic expenses”, 2=“Meeting needs with a little left over” and 3=“Living comfortably”. “Don’t know” and “Refused” responses were included as a separate category.

*Country*. England, Canada, US.

### Analysis

The following analysis was pre-registered prior to data analysis, and analysis code is openly available (https://osf.io/yfbj3/). Participants with missing outcome data (ie, “Refused” to respond) were excluded from the analytic sample. All analyses were conducted in R Version 4.2.3. We present sample characteristics overall and stratified by study condition and country.

For research question 1, we ran a logistic regression predicting no interest in trying, with study condition included (standardised vs branded) as an independent variable. We reported the percentage of participants in each condition with the outcome, and risk ratios (RRs) with 95% confidence intervals (CIs) showing how much more or less likely individuals were to have the outcome in the standardised versus branded condition. Primary models were adjusted for vaping/smoking status, demographics (age group, sex, race/ethnicity, perceived income adequacy) and country. For research question 2, we repeated these analyses with vaping harm perceptions as the outcome.

For research question 3, we repeated the above adjusted (for the same covariates listed previously) models for each outcome, but included interaction terms between condition and age (eg, age*condition), sex, vaping/smoking status, perceived income adequacy and country. Each of these potential interactions with the study conditions was tested in a separate model. We reported the interaction as a ratio of risk ratios (RRRs) alongside 95% CIs. We also reported the RRs and 95% CIs for the intervention effect in each subgroup.

*Sensitivity analyses* We repeated the analysis for research question 1, using the outcome of interest in trying vapes coded as ordinal, as described earlier. Ordinal logistic regression was used to predict interest in trying (one- to six-point ordinal scale) from study condition (standardised vs branded). We reported odds ratios (ORs) with 95% CIs for the standardised versus branded condition. We repeated analyses for research question 2, with the outcome coded as perceiving the vape displayed as not harmful at all (vs otherwise).

### Changes from protocol

Several effects (eg, adjusted models for interest in vaping) could not be estimated using log-binomial regression because the models did not converge. Therefore, we instead calculated marginal risk ratios using logistic regression alongside the *marginal effects* package. 

## Results

### Sample characteristics

Of the 15 479 young people surveyed, 15 259 (98.6%) had complete data on both outcomes, with 188 refusals for interest in trying (75 in branded and 113 in standardised condition) and 50 refusals for harm perceptions (27 in branded and 23 in standardised condition). There were no missing covariate data. Of participants who provided complete outcome data, a total of 7638 were randomised to the branded condition and 7621 to the standardised condition. The CONSORT flow diagram is available in online supplemental figure 1. Sample characteristics are shown in table 1 (by country in online supplemental table 1).

**Table 1:** Sample characteristics, overall and by randomised condition

Characteristic	Overall (N=15 259) n (%)	Branded (N=7638) n (%)	Standardised (N=7621) n (%)
Age (years)			
16–17	5589 (36.6)	2819 (36.9)	2770 (36.3)
18–20	6924 (45.4)	3463 (45.3)	3461 (45.4)
20–29	2746 (18.0)	1356 (17.8)	1390 (18.2)
Sex at birth			
Male	5329 (34.9)	2708 (35.5)	2621 (34.4)
Female	9930 (65.1)	4930 (64.5)	5000 (65.)
Ethnicity			
White	9191 (60.2)	4619 (60.5)	4572 (60.0)
Other	6068 (39.8)	3019 (39.5)	3049 (40.0)
Country			
Canada	5075 (33.3)	2542 (33.3)	2533 (33.2)
England	5151 (33.8)	2587 (33.9)	2564 (33.6)
United States	5033 (33.0)	2509 (32.8)	2524 (33.1)
Vaping/smoking			
Never smoked or vaped	6846 (44.9)	3422 (44.8)	3424 (44.9)
Ever smoked/vaped but not past 30 days	3898 (25.5)	1953 (25.6)	1945 (25.5)
Smoked and vaped in past 30 days	1507 (9.9)	746 (9.8)	761 (10.0)
Only smoked in past 30 days	836 (5.5)	411 (5.4)	425 (5.6)
Only vaped in past 30 days	2172 (14.2)	1106 (14.5)	1066 (14.0)
Perceived income adequacy			
Not meeting basic expenses	959 (6.3)	472 (6.2)	487 (6.4)
Just meeting basic expenses	4647 (30.5)	2339 (30.6)	2308 (30.3)
Meeting needs with a little left over	4954 (32.5)	2524 (33.0)	2430 (31.9)
Living comfortably	3984 (26.1)	1960 (25.7)	2024 (26.6)
Don't know/Refused	715 (4.7)	343 (4.5)	372 (4.9)

### Interest in vaping

Standardisation increased the percentage of participants reporting no interest in trying at least one of the vapes displayed: 67.1% (5112/7621) of participants randomised to view the white standardised devices reported no interest in trying any of the devices shown, compared with 62.8% (4797/ 7638) of those in the branded condition. In the primary covariate-adjusted analysis, standardisation increased the likelihood of reporting no interest in any of the vaping products by 12.7% (adjusted risk ratio (ARR) 1.127, 95% CI 1.085 to 1.169).

**Figure 2:**
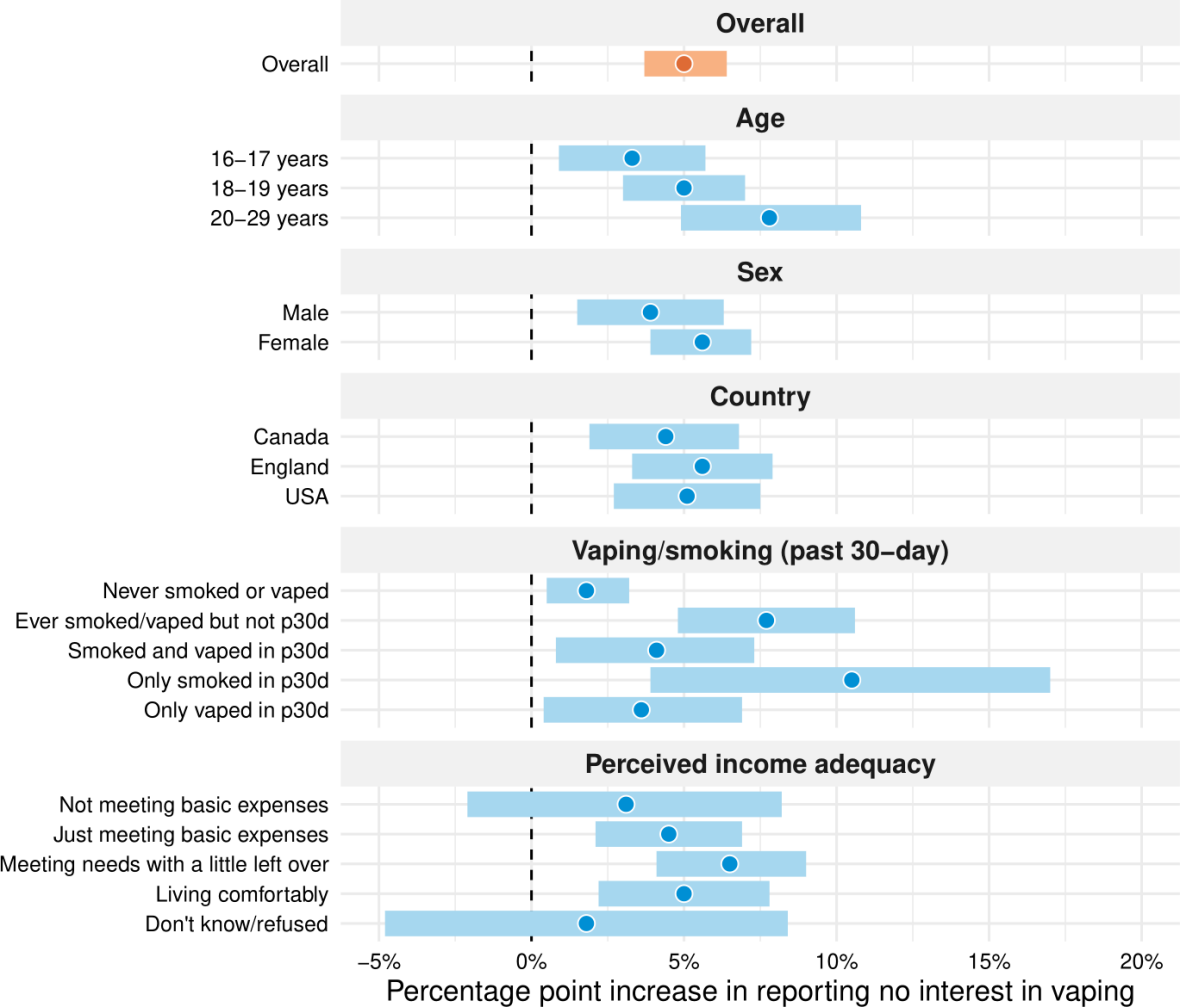
Effect of standardisation on the percentage of young people (aged 16–29 years) reporting no interest in trying vaping, overall and within each subgroup. Participants were randomised to view either four branded (N=7638) or four standardised (N=7621) vape devices. A marginal percentage point increase in participants reporting no interest in trying the vapes was calculated from adjusted models shown in table 2, with shaded bands representing 95% confidence intervals. p30d, past 30 days.

Figure 2 shows the effect of device standardisation within subgroups (more details of these moderation effects are provided in table 2). Standardisation had an effect on all vaping and smoking subgroups, but it was smallest in those who had never vaped or smoked: 92.9% (3182/3424) of those randomised to view standardised white devices reported no interest in trying the vapes compared with 91.2% (3121/3422) of those in the branded condition (ARR 1.020, 95% CI 1.005 to 1.035). Standardisation also had a smaller effect in younger participants compared with older participants (as shown in figure 2 and table 2).

**Table 2:** Overall and subgroup-specific effects of standardising vape devices on no interest in trying vaping

Subgroup	No interest in vaping		Unadjusted effect				Adjusted effect			
	Branded % (n/N)*	Standardised % (n/N)*	RR†	95% CI	Ratio of RR†	95% CI	ARR†	95% CI	Ratio of ARR†	95% CI
Overall	62.8 (4797/7638)	67.1 (5112/7621)	1.068	1.043 to 1.093			1.127	1.085 to 1.169		
By age (years)										
16–17	72.8 (2053/2819)	74.8 (2073/2770)	1.028	0.996 to 1.060	Ref	Ref	1.070	1.017 to 1.124	Ref	Ref
18–19	59.7 (2066/3463)	64.8 (2243/3461)	1.086	1.046 to 1.126	1.057	1.006 to 1.108	1.118	1.068 to 1.167	1.044	0.975 to 1.114
20–29	50.0 (678/1356)	57.3 (796/1390)	1.145	1.065 to 1.226	1.115	1.029 to 1.200	1.187	1.109 to 1.264	1.109	1.018 to 1.200
By sex at birth										
Male	67.1 (1816/2708)	70.2 (1841/2621)	1.047	1.009 to 1.085	Ref	Ref	1.084	1.030 tp 1.139	Ref	Ref
Female	60.5 (2981/4930)	65.4 (3271/5000)	1.082	1.049 to 1.115	1.033	0.984 to 1.082	1.133	1.091 to 1.174	1.044	0.979 to 1.109
By country										
Canada	68.1 (1732/2542)	72.1 (1827/2533)	1.059	1.021 to 1.097	Ref	Ref	1.096	1.040 to 1.152	Ref	Ref
England	55.0 (1422/2587)	59.4 (1522/2564)	1.080	1.029 to 1.131	1.020	0.959 to 1.081	1.135	1.077 to 1.194	1.036	0.961 to 1.111
United States	65.5 (1643/2509)	69.8 (1763/2524)	1.067	1.026 to 1.107	1.008	0.955 to 1.061	1.114	1.057 to 1.171	1.016	0.943 to 1.089
By vaping/smoking										
Never smoked or vaped	91.2 (3121/3422)	92.9 (3182/3424)	1.019	1.005 to 1.033	Ref	Ref	1.020	1.005 to 1.035	Ref	Ref
Ever smoked/vaped but not past 30 days	65.0 (1270/1953)	72.6 (1413/1945)	1.117	1.070 to 1.165	1.096	1.047 to 1.145	1.119	1.071 to 1.167	1.097	1.047 to 1.146
Smoked and vaped in past 30 days	9.5 (71/746)	13.5 (103/761)	1.422	1.017 to 1.827	1.396	0.997 to 1.794	1.420	1.017 to 1.822	1.392	0.997 to 1.787
Only smoked in past 30 days	37.5 (154/411)	47.5 (202/425)	1.268	1.066 to 1.471	1.245	1.045 to 1.445	1.287	1.079 to 1.495	1.262	1.057 to 1.466
Only vaped in past 30 days	16.4 (181/1106)	19.9 (212/1066)	1.215	0.997 to 1.434	1.193	0.978 to 1.407	1.220	1.002 to 1.438	1.196	0.982 to 1.410
By perceived income adequacy										
Not meeting basic expenses	48.1 (227/472)	51.7 (252/487)	1.076	0.939 to 1.213	Ref	Ref	1.073	0.946 to 1.199	Ref	Ref
Just meeting basic expenses	58.2 (1362/2339)	62.5 (1443/2308)	1.074	1.024 to 1.124	0.998	0.863 to 1.133	1.103	1.045 to 1.162	1.029	0.896 to 1.161
Meeting needs with a little left over	64.4 (1625/2524)	69.7 (1694/2430)	1.083	1.040 to 1.125	1.006	0.873 to 1.140	1.148	1.089 to 1.207	1.071	0.933 to 1.208
Living comfortably	68.7 (1347/1960)	72.6 (1470/2024)	1.057	1.014 to 1.099	0.982	0.851 to 1.113	1.109	1.045 to 1.173	1.034	0.899 to 1.170
Don’t know/Refused	68.8 (236/343)	68.0 (253/372)	0.988	0.890 to 1.087	0.919	0.770 to 1.067	1.040	0.893 to 1.186	0.969	0.792 to 1.147

* n (%)=number and percentage who reported no interest in trying vaping. N=total number of participants.

† RR=risk ratio, with branded condition as the reference category. Ratio of RR=ratio of risk ratios with effects (ie, interaction or moderation effects). Marginal RR and ratio of RR calculated from logistic regression models, including an interaction between randomised condition and the potential moderator (with each moderator tested in a separate model).

ARR, adjusted risk ratio; CI, confidence interval; Ref, reference; RR, risk ratio.

In sensitivity analyses using an ordinal outcome, interest in vaping was lower in the standardised condition compared with the branded condition (adjusted odds ratio (AOR) 0.747, 95% CI 0.693 to 0.804) as shown in online supplemental figure 2.

### Harm perceptions

Standardisation did not have a large effect on participants’ harm perceptions of the vaping product displayed: 31.2% (2377/7621) of those randomised to the standardised condition viewed the vape as less harmful than smoking compared with 32.7% (2496/7638) in the branded condition. In primary covariate-adjusted models, the effect of standardisation on harm perceptions was non-significant, but we could not rule out the possibility of small effects (ARR 0.960, 95% CI 0.919 to 1.001). Table 3 shows that there were no clear moderators of the effect of standardisation on harm perceptions.

**Table 3:** Overall and subgroup-specific effects of standardising vape devices on harm perceptions

Subgroup	Perceive vaping as less harmful than smoking		Unadjusted effect				Adjusted effect			
	Branded % (n/N)*	Standardised % (n/N)*	RR†	95% CI	Ratio of RR†	95% CI	ARR†	95% CI	Ratio of ARR†	95% CI
Overall	32.7 (2496/7638)	31.2 (2377/7621)	0.954	0.910 to 0.999			0.960	0.919 to 1.001		
By age (years)										
16–17	28.4 (802/2819)	27.8 (771/2770)	0.978	0.896 to 1.060	Ref	Ref	0.975	0.901 to 1.049	Ref	Ref
18–19	34.5 (1196/3463)	32.2 (1114/3461)	0.932	0.870 to 0.994	0.953	0.851 to 1.055	0.945	0.886 to 1.004	0.969	0.874 to 1.064
20–29	36.7 (498/1356)	35.4 (492/1390)	0.964	0.868 to 1.060	0.985	0.857 to 1.113	0.971	0.878 to 1.063	0.996	0.874 to 1.117
By sex at birth										
Male	33.1 (897/2708)	31.1 (815/2621)	0.939	0.865 to 1.012	Ref	Ref	0.942	0.875 to 1.010	Ref	Ref
Female	32.4 (1599/4930)	31.2 (1562/5000)	0.963	0.908 to 1.019	1.026	0.926 to 1.126	0.970	0.917 to 1.022	1.029	0.937 to 1.121
By country										
Canada	28.9 (734/2542)	26.3 (666/2533)	0.911	0.829 to 0.992	Ref	Ref	0.925	0.849 to 1.001	Ref	Ref
England	44.1 (1140/2587)	43.2 (1107/2564)	0.980	0.919 to 1.041	1.076	0.959 to 1.193	0.976	0.918 to 1.033	1.055	0.948 to 1.162
United States	24.8 (622/2509)	23.9 (604/2524)	0.965	0.871 to 1.059	1.060	0.920 to 1.200	0.975	0.886 to 1.064	1.055	0.925 to 1.184
By vaping/smoking										
Never smoked or vaped	23.8 (815/3422)	21.8 (747/3424)	0.916	0.836 to 0.996	Ref	Ref	0.924	0.844 to 1.003	Ref	Ref
Ever smoked/vaped but not p30d	33.8 (661/1953)	31.3 (608/1945)	0.924	0.840 to 1.007	1.008	0.882 to 1.135	0.920	0.837 to 1.002	0.996	0.872 to 1.119
Smoked and vaped in p30d	45.4 (339/746)	48.8 (371/761)	1.073	0.958 to 1.188	1.171	1.009 to 1.333	1.079	0.957 to 1.202	1.169	1.002 to 1.335
Only smoked in p30d	30.9 (127/411)	31.5 (134/425)	1.020	0.815 to 1.226	1.114	0.869 to 1.358	1.021	0.814 to 1.228	1.105	0.862 to 1.349
Only vaped in p30d	50.1 (554/1106)	48.5 (517/1066)	0.968	0.886 to 1.051	1.057	0.928 to 1.186	0.971	0.88 to 1.055	1.051	0.923 to 1.179
By perceived income adequacy										
Not meeting basic expenses	33.1 (156/472)	29.8 (145/487)	0.901	0.732 to 1.070	Ref	Ref	0.906	0.748 to 1.064	Ref	Ref
Just meeting basic expenses	33.4 (782/2339)	31.7 (731/2308)	0.947	0.869 to 1.026	1.052	0.836 to 1.267	0.952	0.880 to 1.025	1.051	0.851 to 1.251
Meeting needs with a little left over	34.9 (882/2524)	32.4 (787/2430)	0.927	0.854 to 0.999	1.029	0.820 to 1.238	0.935	0.870 to 1.001	1.033	0.839 to 1.227
Living comfortably	30.6 (599/1960)	31.2 (631/2024)	1.020	0.925 to 1.115	1.132	0.896 to 1.369	1.016	0.933 to 1.099	1.122	0.905 to 1.338
Don’t know/Refused	22.4 (77/343)	22.3 (83/372)	0.994	0.722 to 1.265	1.103	0.738 to 1.469	1.006	0.754 to 1.257	1.110	0.771 to 1.449

* n (%)=number and percentage who perceived the vape shown as less harmful than smoking. N=total number of participants.

† RR=risk ratio, with branded condition as the reference category. Ratio of RR=ratio of risk ratios with effects (ie, interaction or moderation effects). Marginal RR and ratio of RR calculated from logistic regression models, including an interaction between randomised condition and the potential moderator (with each moderator tested in a separate model).

ARR, adjusted risk ratio; CI, confidence interval; p30d, past 30 days; Ref, reference; RR, risk ratio.

In sensitivity analyses, similarly low proportions of participants in both conditions perceived the displayed vaping device as not at all harmful: 2.57% (196/7621) in the standardised versus 2.19% (167/7638) in the branded condition (ARR 1.176, 95% CI 0.946 to 1.407). The full distribution of harm perceptions is provided in online supplemental table 2.

Sensitivity analyses were also conducted examining moderation effects when all two-way interactions were included in a single model; results using this approach were analogous to those when examining each moderator in a separate model (online supplemental tables 3 and 4).

## Discussion

### Summary of findings

As hypothesised, in this randomised experiment of 15 259 16–29-year-olds in England, Canada and the US, we found that standardising the colour and branding of disposable vaping devices increased the percentage who were not interested in trying any of the vaping products from 63% to 67%, with minimal adverse impact on harm perceptions. Standardisation increased those uninterested in all vaping and smoking subgroups, but the greatest impact was among those who had smoked and/or vaped in the past 30 days. For example, standardisation increased the percentage with no interest in trying any vape from 38% to 48% among those who had smoked but not vaped in the past 30 days, individuals who would gain from switching entirely from smoking to vaping. Among those who had never smoked or vaped, standardisation also increased those not interested in trying, although to a lesser extent. The vast majority of those who never smoked or vaped reported no interest in trying the vapes, whether they were branded (91.2%) or standardised (92.9%). Standardisation also increased those uninterested in those who exclusively vaped in the past 30 days (16.4% reported no interest in trying the branded devices vs 19.9% for the standardised devices), a group who would benefit from stopping vaping. The effect of standardisation on reducing interest in vaping was also smallest in the youngest age group, among whom most had never vaped or smoked. There was no clear evidence of differences in the effect of standardisation by sex, country (Canada, England, US) or perceived income adequacy.

### Interpretation and comparison with prior literature

Our findings are broadly consistent with prior literature on e-cigarette product packaging that suggests that standardisation reduces appeal among young people,^21–23 31^ and similar to literature on tobacco cigarettes showing that standardised cigarette sticks are less appealing to youth.^25^ However, inconsistent with prior work,^22^ we found greater effects of standardisation on interest among young adults compared with youth and among participants who vaped and/or smoked compared with those who had never done so. These differences may reflect differing samples; prior work has compared youth aged 11–18 years with all adults aged 18+ years while we compared 16–17-year-olds with young adults aged 18–29 years. Finally, we only examined effects of standardisation among youth and young adults. It is possible that the impact of standardisation would not generalise to other groups; it will be important for subsequent studies to examine effects in older age groups, especially those who smoke.

The smaller effect identified among people who have never vaped or smoked does not necessarily mean that standardisation will fail to discourage uptake; there may have been a ceiling effect associated with the outcome measure, given that over 90% of never-users reported no interest in any of the vaping products presented. Future studies could usefully look at the impact on later uptake or use measures that capture varying levels of interest.

Importantly, our findings suggest little to no effect of standardising devices on relative harm perceptions. Relative harm perceptions of vapes are often inaccurate among youth and adults, and this can influence the likelihood of someone using a vape to quit.^13 32 33^ Therefore, it is promising that standardising the appearance of devices to be white and unbranded would likely reduce appeal without unintentionally exacerbating misperceptions (although there may have been greater impact on harm perceptions if the colour of standardisation was brown not white).^34^

### Policy implications

In January 2024, the UK government announced plans to introduce new measures to ensure that e-cigarette manufacturers implement standardised packaging, but it remains unclear exactly what standardised packaging would look like.^20^ Our findings suggest that the integration of regulation on device design into new policy further reduces the appeal of vapes to young people. However, compared with people who have never smoked or vaped, the reduction in interest was more pronounced among smokers, who might benefit from using vapes to quit smoking. There is a risk that the public health benefits of preventing youth uptake of vaping could be offset by a decline in the number of young people transitioning from smoking to exclusive vaping, or an increase in relapse from exclusive vaping to smoking, including dual use.^10 35^ Future research should explore how standardisation impacts these transition pathways between exclusive smoking, exclusive vaping and dual use. Nonetheless, if legislation on standardised vaping devices is introduced, any unintended consequences could be mitigated through complementary policies aimed at promoting smoking cessation and preventing relapse among former smokers.

### Strengths and limitations

Strengths of this study include the large sample size, to precisely estimate the effect of standardisation overall and across important subgroups (eg, vaping/smoking status), use of a randomised controlled experiment to avoid confounding, and consistency of results across three countries with differing vaping markets and regulations.

There were also limitations. First, as mentioned earlier, our binary measure of interest (no interest vs other) in vaping may have been subject to a ceiling effect, making it insensitive to detecting differences in appeal of vapes to people who have never vaped or smoked. Future studies should replicate results using a wider array of measures, including — if possible — measures that capture people’s automatic cognitive processing of packaging (eg, eye tracking) as well as conscious reporting of interest and appeal. Second, interest in vaping was self-reported, yet people do not have perfect insight into the factors of products that drive them to use them.^36^ Third, an online experimental paradigm lacks ecological validity^1 37^; it may not reflect an individual's interest if they encountered these products in a more naturalistic setting, such as when offered by a friend or viewing a product in a store among a wider range of competing products. However, at least for cigarettes, experimental studies and real-world evaluations of the impact of standardised packaging have found highly consistent results.^16 38^ Fourth, the low rates of reporting no interest among people who both smoke and vape may have limited our power to detect any genuine difference between this group and people who have never smoked or vaped. Fifth, different provinces and countries have different ages of sale for vaping products. Future research could examine what effect this has on interest in vaping. Finally, the wording of the interest in trying vaping measure may have influenced responses by prompting participants to report interest in trying at least one product. However, the low interest rates (<10%) among people who have never smoked or vaped suggest that participants were comfortable reporting no interest. As this potential demand effect would have applied equally to both study conditions, it is unlikely to have biased the observed differences between standardised and branded packaging.

## Conclusions

Standardising the colour and branding of vaping devices reduces interest in trying them among 16–29-year-olds across all vaping and smoking subgroups, but with the effects strongest among those who smoke and/or vape. Effects on harm perceptions of vaping were minimal. For countries interested in discouraging vaping among young people, standardising the colour of vaping devices could be considered alongside standardised packaging as a potential policy option. However, there may be unintended consequences in terms of discouraging those who smoke from switching to vaping, which should be further investigated and possibly balanced with other targeted policies to encourage smoking cessation.

## Supplementary material

10.1136/tc-2024-059210 online supplemental file 1

## Data Availability

Data are available upon reasonable request.
