# Supplementary material for: Impact of standardising the colour and branding of vape devices on product appeal among young people: a randomised experiment in England, Canada and the United States
Source: Tob Control. 2025 May 20;35(4):e059210. doi: 10.1136/tc-2024-059210 (PMC12353783; doi:10.1136/tc-2024-059210)
Supplement: Supplementary file 1 [file tc-35-4-s001.pdf]

# Supplementary Tables

## Supplementary Table 1. Sample characteristics in Canada, England and the US, overall and by randomised condition.

| | *Canada* | | | *England* | | | *US* | | |
|---|---|---|---|---|---|---|---|---|---|
| **Characteristic** | **Overall,** ***N = 5,075***[1] | **Branded,** ***N = 2,542***[1] | **Standardised,** ***N = 2,533***[1] | **Overall,** ***N = 5,151***[1] | **Branded,** ***N = 2,587***[1] | **Standardised,** ***N = 2,564***[1] | **Overall,** ***N = 5,033***[1] | **Branded,** ***N = 2,509***[1] | **Standardised,** ***N = 2,524***[1] |
| **Age (years)** | | | | | | | | | |
| 16-17 | 1,948 (38.4%) | 980 (38.6%) | 968 (38.2%) | 1,776 (34.5%) | 892 (34.5%) | 884 (34.5%) | 1,865 (37.1%) | 947 (37.7%) | 918 (36.4%) |
| 18-20 | 2,207 (43.5%) | 1,109 (43.6%) | 1,098 (43.3%) | 2,454 (47.6%) | 1,226 (47.4%) | 1,228 (47.9%) | 2,263 (45.0%) | 1,128 (45.0%) | 1,135 (45.0%) |
| 20-29 | 920 (18.1%) | 453 (17.8%) | 467 (18.4%) | 921 (17.9%) | 469 (18.1%) | 452 (17.6%) | 905 (18.0%) | 434 (17.3%) | 471 (18.7%) |
| **Sex** | | | | | | | | | |
| Male | 1,905 (37.5%) | 968 (38.1%) | 937 (37.0%) | 1,768 (34.3%) | 898 (34.7%) | 870 (33.9%) | 1,656 (32.9%) | 842 (33.6%) | 814 (32.3%) |
| Female | 3,170 (62.5%) | 1,574 (61.9%) | 1,596 (63.0%) | 3,383 (65.7%) | 1,689 (65.3%) | 1,694 (66.1%) | 3,377 (67.1%) | 1,667 (66.4%) | 1,710 (67.7%) |
| **Ethnicity** | | | | | | | | | |
| White | 2,905 (57.2%) | 1,447 (56.9%) | 1,458 (57.6%) | 3,467 (67.3%) | 1,772 (68.5%) | 1,695 (66.1%) | 2,819 (56.0%) | 1,400 (55.8%) | 1,419 (56.2%) |
| Other | 2,170 (42.8%) | 1,095 (43.1%) | 1,075 (42.4%) | 1,684 (32.7%) | 815 (31.5%) | 869 (33.9%) | 2,214 (44.0%) | 1,109 (44.2%) | 1,105 (43.8%) |
| **Vaping/smoking**[2] | | | | | | | | | |
| Never smoked or vaped | 2,491 (49.1%) | 1,244 (48.9%) | 1,247 (49.2%) | 1,927 (37.4%) | 983 (38.0%) | 944 (36.8%) | 2,428 (48.2%) | 1,195 (47.6%) | 1,233 (48.9%) |
| Ever but not p30d | 1,350 (26.6%) | 672 (26.4%) | 678 (26.8%) | 1,259 (24.4%) | 624 (24.1%) | 635 (24.8%) | 1,289 (25.6%) | 657 (26.2%) | 632 (25.0%) |
| Smoked and vaped in p30d | 336 (6.6%) | 166 (6.5%) | 170 (6.7%) | 792 (15.4%) | 400 (15.5%) | 392 (15.3%) | 379 (7.5%) | 180 (7.2%) | 199 (7.9%) |
| Only smoked in p30d | 303 (6.0%) | 149 (5.9%) | 154 (6.1%) | 303 (5.9%) | 146 (5.6%) | 157 (6.1%) | 230 (4.6%) | 116 (4.6%) | 114 (4.5%) |
| Only vaped in p30d | 595 (11.7%) | 311 (12.2%) | 284 (11.2%) | 870 (16.9%) | 434 (16.8%) | 436 (17.0%) | 707 (14.0%) | 361 (14.4%) | 346 (13.7%) |
| **Perceived income adequacy** | | | | | | | | | |
| Not meeting basic expenses | 262 (5.2%) | 130 (5.1%) | 132 (5.2%) | 264 (5.1%) | 127 (4.9%) | 137 (5.3%) | 433 (8.6%) | 215 (8.6%) | 218 (8.6%) |
| Just meeting basic expenses | 1,417 (27.9%) | 721 (28.4%) | 696 (27.5%) | 1,609 (31.2%) | 802 (31.0%) | 807 (31.5%) | 1,621 (32.2%) | 816 (32.5%) | 805 (31.9%) |
| Meeting needs with a little left over | 1,724 (34.0%) | 872 (34.3%) | 852 (33.6%) | 1,728 (33.5%) | 891 (34.4%) | 837 (32.6%) | 1,502 (29.8%) | 761 (30.3%) | 741 (29.4%) |
| Living comfortably | 1,458 (28.7%) | 723 (28.4%) | 735 (29.0%) | 1,304 (25.3%) | 637 (24.6%) | 667 (26.0%) | 1,222 (24.3%) | 600 (23.9%) | 622 (24.6%) |
| Don't know/refused | 214 (4.2%) | 96 (3.8%) | 118 (4.7%) | 246 (4.8%) | 130 (5.0%) | 116 (4.5%) | 255 (5.1%) | 117 (4.7%) | 138 (5.5%) |

[1] n (%)
[2] p30d = past 30 days

**Supplementary Table 2. Vaping harm perceptions in young people (16-29 years) randomised to view either one randomly selected branded or standardised vape device[1].**

| Harm perception of vape shown | Branded, *N = 7,638*[2] | Standardised, *N = 7,621*[2] |
|---|---|---|
| Not at all harmful | 2.2% (167) | 2.6% (196) |
| Harmful, but less harmful than smoking cigarettes | 30.5% (2329) | 28.6% (2181) |
| As harmful as smoking cigarettes | 44.4% (3395) | 44.4% (3381) |
| More harmful than smoking cigarettes | 11.2% (854) | 11.8% (898) |
| Don't know | 11.7% (893) | 12.7% (965) |

[1] Within each randomised condition, each participant viewed one vape randomly selected from the four options (see Figure 1) and was asked "How harmful do you think it is to vape this product?".
[1] % (N)

**Supplementary Table 3. Moderation effects for impact of standardising vape devices on no interest in vaping estimated in (1) separate models for each moderator or (2) a single model that includes all two-way interactions.**

| | 1. Separate models | | 2. All two-way interactions | |
|---|---|---|---|---|
| | Ratio of ARR[1] | 95%CI[1] | Ratio of ARR[1] | 95%CI[1] |
| **OVERALL** | | | | |
| **BY AGE** | | | | |
| 16-17 years | Ref | Ref | Ref | Ref |
| 18-19 years | 1.044 | 0.975-1.114 | 1.037 | 0.999-1.076 |
| 20-29 years | 1.109 | 1.018-1.200 | 1.116 | 1.049-1.182 |
| **BY SEX AT BIRTH** | | | | |
| Male | Ref | Ref | Ref | Ref |
| Female | 1.044 | 0.979-1.109 | 1.033 | 0.996-1.070 |
| **BY COUNTRY** | | | | |
| Canada | Ref | Ref | Ref | Ref |
| England | 1.036 | 0.961-1.111 | 1.037 | 0.990-1.083 |
| US | 1.016 | 0.943-1.089 | 1.011 | 0.970-1.052 |
| **BY VAPING/SMOKING**[2] | | | | |
| Never smoked or vaped | Ref | Ref | Ref | Ref |
| Ever smoked/vaped but not p30d | 1.097 | 1.047-1.146 | 1.096 | 1.047-1.145 |
| Smoked and vaped in p30d | 1.392 | 0.997-1.787 | 1.394 | 0.998-1.791 |
| Only smoked in p30d | 1.262 | 1.057-1.466 | 1.258 | 1.057-1.458 |
| Only vaped in p30d | 1.196 | 0.982-1.410 | 1.197 | 0.983-1.412 |
| **BY PERCEIVED INCOME ADEQUACY** | | | | |
| Not meeting basic expenses | Ref | Ref | Ref | Ref |
| Just meeting basic expenses | 1.029 | 0.896-1.161 | 1.009 | 0.906-1.112 |
| Meeting needs with a little left over | 1.071 | 0.933-1.208 | 1.024 | 0.921-1.126 |
| Living comfortably | 1.034 | 0.899-1.170 | 0.998 | 0.898-1.098 |
| Don’t know/refused | 0.969 | 0.792-1.147 | 0.964 | 0.847-1.081 |

ARR = Adjusted risk ratio

**Supplementary Table 4. Moderation effects for impact of standardising vape devices on harm perceptions estimated in (1) separate models for each moderator or (2) a single model that includes all two-way interactions.**

| | 1. Separate models | | 2. All two-way interactions | |
|---|---|---|---|---|
| | Ratio of ARR[1] | 95%CI[1] | Ratio of ARR[1] | 95%CI[1] |
| **OVERALL** | | | | |
| **BY AGE** | | | | |
| 16-17 years | Ref | Ref | Ref | Ref |
| 18-19 years | 0.969 | 0.874-1.064 | 0.970 | 0.870-1.070 |
| 20-29 years | 0.996 | 0.874-1.117 | 0.997 | 0.872-1.123 |
| **BY SEX AT BIRTH** | | | | |
| Male | Ref | Ref | Ref | Ref |
| Female | 1.029 | 0.937-1.121 | 1.032 | 0.936-1.128 |
| **BY COUNTRY** | | | | |
| Canada | Ref | Ref | Ref | Ref |
| England | 1.055 | 0.948-1.162 | 1.059 | 0.947-1.172 |
| US | 1.055 | 0.925-1.184 | 1.058 | 0.922-1.195 |
| **BY VAPING/SMOKING**[2] | | | | |
| Never smoked or vaped | Ref | Ref | Ref | Ref |
| Ever smoked/vaped but not p30d | 0.996 | 0.872-1.119 | 0.997 | 0.873-1.120 |
| Smoked and vaped in p30d | 1.169 | 1.002-1.335 | 1.163 | 1.005-1.321 |
| Only smoked in p30d | 1.105 | 0.862-1.349 | 1.105 | 0.866-1.344 |
| Only vaped in p30d | 1.051 | 0.923-1.179 | 1.053 | 0.926-1.179 |
| **BY PERCEIVED INCOME ADEQUACY** | | | | |
| Not meeting basic expenses | Ref | Ref | Ref | Ref |
| Just meeting basic expenses | 1.051 | 0.851-1.251 | 1.052 | 0.845-1.260 |
| Meeting needs with a little left over | 1.033 | 0.839-1.227 | 1.031 | 0.829-1.233 |
| Living comfortably | 1.122 | 0.905-1.338 | 1.127 | 0.900-1.354 |
| Don’t know/refused | 1.110 | 0.771-1.449 | 1.114 | 0.758-1.471 |

ARR = Adjusted risk ratio

## Supplementary Figures

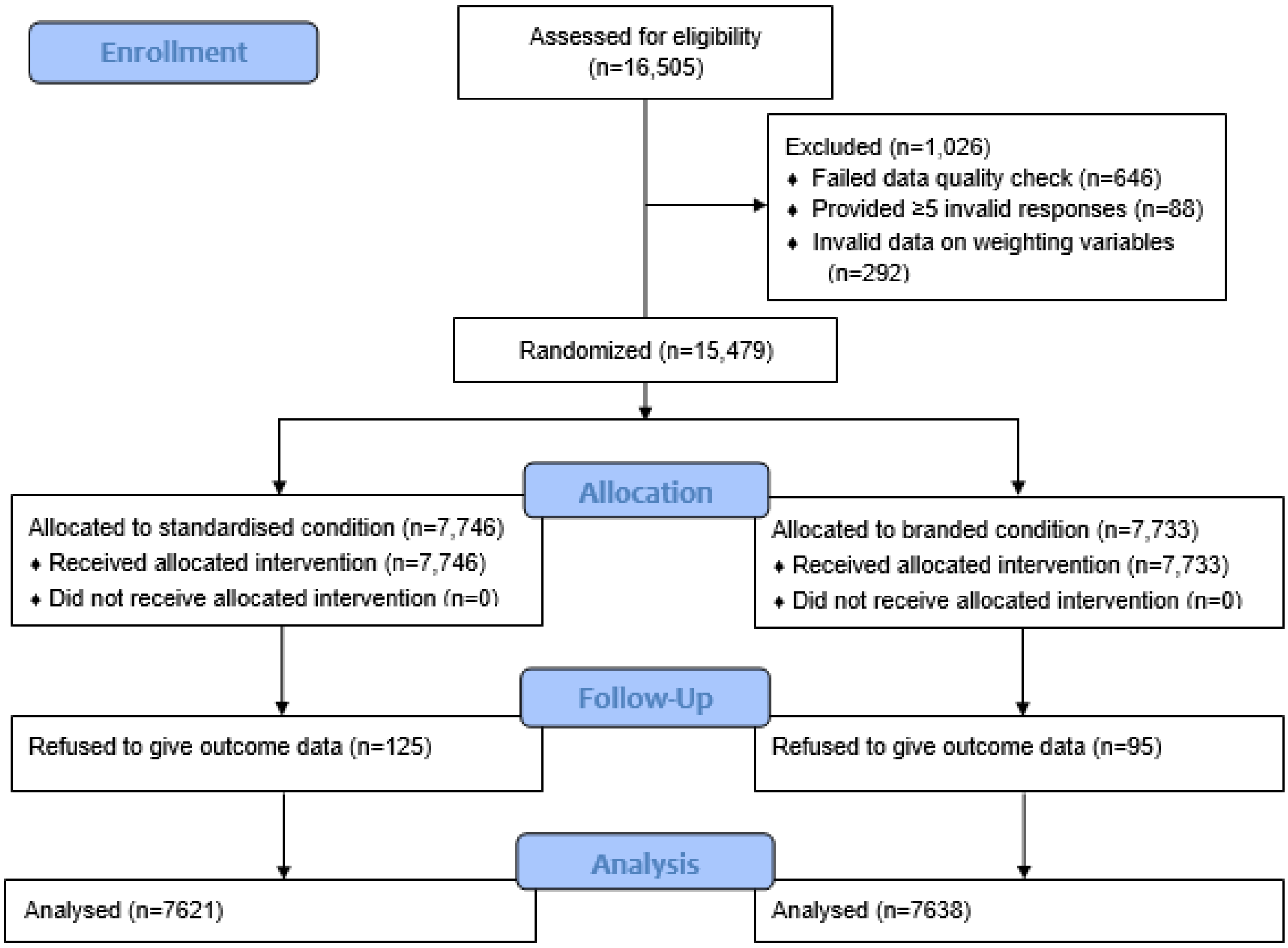


**Supplementary Figure 1. CONSORT 2010 Flow diagram showing participants enrolled, randomly allocated, followed-up, and analysed.**

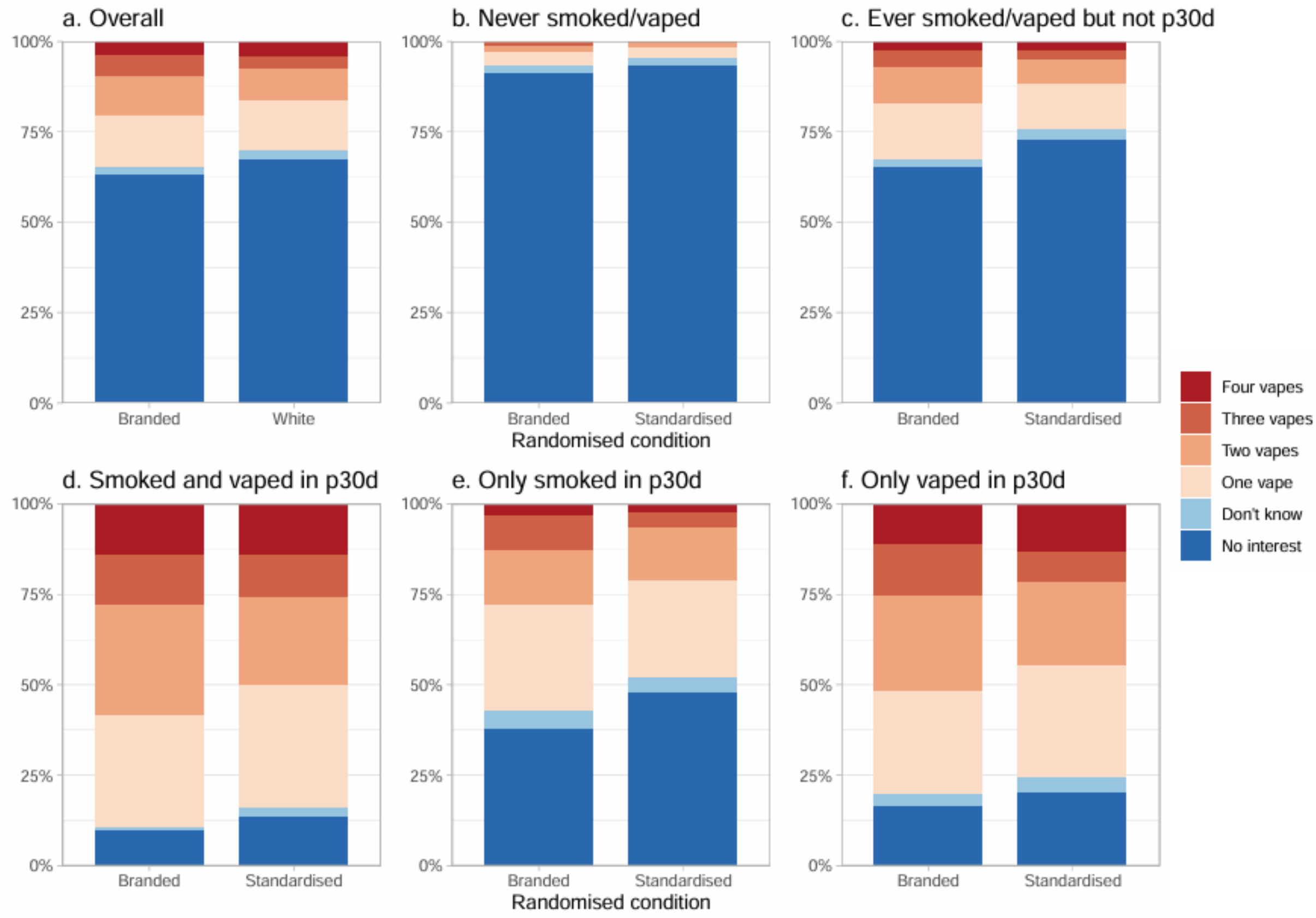


**Supplementary Figure 2. Interest in trying vapes among young people (16-29 years) randomised to view either four branded (*N*=7638) or four standardised (*N*=7621) vape devices, overall and stratified by smoking/vaping status.** Ordinal logistic regression analyses showed that standardisation reduced interest in vaping overall (adjusted odds ratio [AOR=0.75, 95%CI=0.69-0.80, p<.00001) and in those who: had never smoked or vaped (AOR=0.77, 95%CI=0.65-0.92, p=.004), had ever smoked or vaped but not in the past 30 days (AOR=0.69, 95%CI=0.60-0.78, p<.00001), both smoked and vaped in the past 30 days (AOR=0.78, 95%CI=0.65-0.94, p=.009), only smoked in past 30 days (AOR=0.67, 95%CI=0.52-0.87, p=.002), and only vaped in the past 30 days (AOR=0.80, 95%CI=0.69-0.93, p=.003).
